# Life-span of in vitro differentiated *Plasmodium falciparum* gametocytes

**DOI:** 10.1186/s12936-017-1986-6

**Published:** 2017-08-11

**Authors:** Tamirat Gebru, Albert Lalremruata, Peter G. Kremsner, Benjamin Mordmüller, Jana Held

**Affiliations:** 1 Institute of Tropical Medicine, University of Tübingen, Tübingen, Germany; 2 German Centre for Infection Research (DZIF), partner site Tübingen, Tübingen, Germany; 3 Department of Medical Laboratory Sciences, College of Medical and Health Sciences, Haramaya University, Harar, Ethiopia

**Keywords:** Malaria, Clinical isolate, Gametocytogenesis, Gametocyte viability, Gametocyte circulation time, Gametocyte longevity, Exflagellation

## Abstract

**Background:**

The sexual stages (gametocytes) of *Plasmodium falciparum* do not directly contribute to the pathology of malaria but are essential for transmission of the parasite from the human host to the mosquito. Mature gametocytes circulate in infected human blood for several days and their circulation time has been modelled mathematically from data of previous in vivo studies. This is the first time that longevity of gametocytes is studied experimentally in vitro.

**Methods:**

The in vitro longevity of *P. falciparum* gametocytes of 1 clinical isolate and 2 laboratory strains was assessed by three different methods: microscopy, flow cytometry and reverse transcription quantitative real-time PCR (RT-qPCR). Additionally, the rate of gametocytogenesis of the used *P. falciparum* strains was compared.

**Results:**

The maximum in vitro lifespan *of P. falciparum* gametocytes reached almost 2 months (49 days by flow cytometry, 46 days by microscopy, and at least 52 days by RT-qPCR) from the starting day of gametocyte culture to death of last parasite in the tested strains with an average 50% survival rate of 6.5, 2.6 and 3.5 days, respectively. Peak gametocytaemia was observed on average 19 days after initiation of gametocyte culture followed by a steady decline due to natural decay of the parasites. The rate of gametocytogenesis was highest in the NF54 strain.

**Conclusions:**

*Plasmodium falciparum* mature gametocytes can survive up to 16–32 days (at least 14 days for mature male gametocytes) in vitro in absence of the influence of host factors. This confirms experimentally a previous modelling estimate that used molecular tools for gametocyte detection in treated patients. The survival time might reflect the time the parasite can be transmitted to the mosquito after clearance of asexual parasites. These results underline the importance of efficient transmission blocking agents in the fight against malaria.

**Electronic supplementary material:**

The online version of this article (doi:10.1186/s12936-017-1986-6) contains supplementary material, which is available to authorized users.

## Background

Malaria is one of the most important infectious diseases, leading to approximately half a million annual deaths globally [1]. Among the different *Plasmodium* species that cause disease in humans, *Plasmodium falciparum* is the most virulent form and is responsible for most of the malaria morbidity and almost all malaria mortality in sub-Saharan Africa. The parasite is transmitted from the human host to the mosquito through mature sexual stages of the parasite (gametocytes) that circulate in peripheral blood. In the life cycle of *P. falciparum*, a certain percentage of asexual blood-stage parasites develop stochastically to gametocytes [2]. The immature forms (stage I–IV) sequester in internal organs [3, 4] for a maximum of 12 days [2], before developing to mature gametocytes (stage V) that appear in the peripheral circulation and can be taken up by a blood-sucking female *Anopheles* mosquito. Once in the mosquito’s midgut the sexual cycle continues leading to infective sporozoites in the salivary glands that render the mosquito infective to the next person it will bite.

As gametocytes are responsible for bridging the human/vector transmission of the parasite, studying their biology, development and viability or life span is of paramount importance for designing an effective transmission-blocking strategy. Neglected in the past, this field is now getting more attention with a revival of interest for the regional elimination and worldwide eradication of malaria [5, 6].

It has been shown earlier that even sub-microscopic densities of gametocytes in humans can establish mosquito infection [7, 8]. The chance of transmission of the parasite to the mosquito increases with the circulation time of the parasite [9, 10]. Most anti-malarials are not effective against the transmission stages and only act indirectly by reduction of asexual stages or immature gametocytes [11, 12]. After an effective clearance of asexual parasites either by drugs or the immune response, the duration of gametocyte carriage depends on the sequestration and subsequent circulation time of gametocytes in the blood stream before the natural decay of the parasite happens. Once in the circulation, they remain infective for several days and may be even weeks, able to continue the transmission [9], but the exact time span is not known. Mathematical modelling in vivo after clearance of asexual stages by anti-malarial treatment in patients and subsequent detection by microscopy or molecular methods was so far the main method by which longevity of gametocytes was estimated. In these modelling approaches, assumptions were made considering gametocyte conversion probability, sequestration periods and decay of gametocytes [2, 10, 13].

Even though transmission of the parasite to the mosquito is associated with circulation time [14, 15] and density of mature gametocytes [16, 17] in the infected person, this parameter is not well studied as it is technically difficult to measure gametocyte longevity in vivo. The density of gametocytes in the circulation is often below the detection limit of commonly used methods and to follow in vivo a single mature gametocyte when released from the site of sequestration to the peripheral circulation has never been achieved. However, in vitro there is the possibility to follow gametocytes for their life span and evaluate their natural decay. In this study, the longevity of gametocytes was assessed in vitro by three different methods: microscopy, flow cytometry and reverse transcription quantitative real-time polymerase chain reaction (RT qPCR). In addition, the rate of gametocytogenesis was evaluated in the different *P. falciparum* strains by microscopy, to characterize variation in gametocyte development.

## Methods

### Gametocyte culture

Gametocyte culture was initiated from an asexual culture of 1 clinical isolate (JH013) and 2 laboratory strains (3D7 and NF54) of *P. falciparum.* The clinical isolate (JH013) was selected from a previous study conducted in Lambaréné, Gabon [18]. The isolate was collected from a patient presenting with *P. falciparum* mono-infection and preserved in glycerolyte at −196 °C until culturing. JH013 and the *P. falciparum* laboratory strains 3D7 (Malaria Research and Reference Reagent Resource (ATCC, Virginia, USA) and NF54, isolated from a volunteer inoculated with PfSPZ Challenge for controlled human malaria infection (Sanaria Inc, Rockville, MD, USA) [19], were kept in asexual culture at 5% haematocrit in RPMI 1640 supplemented with 25 mM HEPES, 28 mM NaHCO3, 50 μg/mL gentamicin, 0.5% w/v Albumax II, 2.4 mM l-glutamine, and 0.14 mM hypoxanthine at 5% CO_2_ and 5% O_2_. Naïve serum (5%) was added for the cultivation of the asexual parasites of the clinical isolate. The medium was replaced every 24 h.

Gametocyte culture was performed as described before [11] with modifications: the culture was started from asexual parasites that were kept in a continuous culture with sorbitol synchronization twice weekly. The same culture medium was used for the gametocyte development as for the asexual culture except the addition of 5% naïve serum for both clinical and laboratory strains. The culture was initiated with 9% haematocrit and 0.5% parasitaemia in 20 mL total volume and was kept in a 5% O_2_/CO_2_ atmosphere at 37 °C with daily change of medium without parasite dilution. In the second week, the volume of medium was doubled and thereby the haematocrit reduced to 4.5%. On days 11–15 and from day 19 on every 5 days for 2 consecutive days, cultures were treated with 50 mM *N*-acetyl-d-glucosamine (MP Biomedicals GmbH, Santa Ana, CA, USA) to remove or suppress asexual parasites to avoid emergence of new gametocytes. The culture was followed until all gametocytes were judged as dead with daily change of complete medium without any purification or enrichment step.

### Assessment of gametocyte longevity

Microscopy, flow cytometry and RNA-based RT-qPCR were used to assess the longevity of gametocytes of different strains of *P. falciparum*. Day 0 is the starting day of gametocyte culture and the culture was followed until the last positive signal representing a gametocyte detected by the respective technique. Longevity after reaching maturity represents the estimated in vivo circulation time as only mature gametocytes circulate freely in the blood. This was calculated by subtracting the number of days needed to reach peak gametocytaemia (see day of peak gametocytaemia in Table 1). On the day of peak gametocytaemia all gametocytes in the respective culture reached maturity (stage V), as checked by microscopy. The 50 and 10% survival rate of mature gametocytes was also calculated starting with the day of peak parasitaemia. After initiation of gametocytogenesis a sample was taken on days 3, 5, 8, 10, 12, 18, 20, 22, 27, 33, 35, 38, 40, 43, 46, 49, and 52 and analysed by microscopy, flow cytometry and RTqPCR.

**Table 1:** Summary of in vitro gametocyte longevity data evaluated by three viability assays from cultures of one *Plasmodium falciparum* clinical isolate and two laboratory strains

*Plasmodium falciparum*	Gametocyte longevity in days: mean (range)			
	Microscopy	Flow cytometry	RT-qPCR	
			Pfs25	PF14_0367^a^
3D7	–	46 (43–49)	–	–
NF54	46	49 (48–50)	>52	>52
JH013	39 (38–40)	45 (43–49)	>52	46
Average number of days of gametocyte culture to reach peak gametocytaemia (maturation)^c^	20.5	16	19	19
Mature gametocytes 50% survival rate (days)	2.6	6.5	4.5	2.5
Mature gametocytes 10% survival rate (days)	10	12	13.3	10.4
Mature gametocyte circulation time (days)^b^	18–26	16–24	31–32	26–31

^a^Mid and mature gametocyte marker [23]

^b^Circulation time is calculated by subtracting the number of days needed to reach peak gametocytaemia

^c^On the day of peak gametocytaemia all gametocytes in the respective culture have reached maturity (stage V)

### Assessment of gametocyte longevity by microscopy

Viability of gametocytes was assessed microscopically in two parasite strains, the NF54 laboratory strain and JH013 clinical isolate by evaluating the morphology of parasites in a Giemsa-stained thin blood smear. Gametocytes were classified either as viable (normal intact morphology of mature gametocytes) or dead (deformed cells with a decrease in width, a thin needle-like appearance or degraded cytoplasmic content). Parasitaemia of mature gametocytes ((number of mature gametocyte infected erythrocytes/number of erythrocytes) × 100) was determined by two trained and qualified readers blinded to each other and the slide identification (ID). Samples were declared negative if no intact and mature gametocytes were seen in 20,000 erythrocytes. In case of discordant results a third reading was performed.

To verify viability, an exflagellation assay (incubation for 20 min in exflagellation medium supplemented with xanthurenic acid as described earlier [20]) was additionally performed on days 17, 18, 19, 20, 22, 27, 33, 35, and 38 to assess exflagellation of male gametocytes in NF54 and JH013.

### Assessment of gametocyte longevity by flow cytometry

Viability and life span of gametocytes of *P. falciparum* isolates (3D7, NF54, JH013) was assessed also by hydroethidine (HE) staining evaluated by flow cytometry to measure metabolic activity. HE staining has been used earlier in a flow cytometric gametocyte drug assay [21]. HE staining (Polysciences Europe GmbH, Eppelheim, Germany) was performed at a final concentration of 50 µg/mL in medium for 20 min at 37 °C in the dark before analysis by flow cytometry (BD FACS Canto II) with the FACSDiva software version 6.1.2 (BD Biosciences, San Jose, USA). Metabolically active parasites convert HE to ethidium that interacts with the parasites’ nucleic acids showing red fluorescence emission when excited (see panel B of Additional file 1: Figure S1). The result is expressed as the percentage of positive fluorescent cells (PPFC) in 50,000 erythrocytes. As a negative control, uninfected erythrocytes (incubated with medium in the same way as the culture) were used for gating (see panel A of Additional file 1: Figure S1). The gametocyte culture was followed until no positive signal above the negative control could be seen after HE staining. After day 15, a control slide was additionally done and read by microscopy to exclude presence of asexual parasites.

### Assessment of gametocyte longevity by reverse transcription quantitative PCR

#### RNA extraction

On the sampling days, 40 µL of erythrocytes was taken out of the culture for molecular detection of gametocytes and was added to 100 µL Trizol reagent (Life Technologies GmbH, Darmstadt, Germany) for RNA stabilization and stored at −80 °C until RNA extraction. RNA purification was done automated on the QIAsymphony SP (QIAGEN GmbH, Hilden, Germany) using the QIAsymphony RNA Kit (QIAGEN GmbH, Hilden, Germany) and RNA CT 400 protocol provided with the instrument. Before purification, frozen samples were thawed and vortexed followed by an incubation at room temperature (RT) for 5 min. Then 20 µL chloroform (Sigma-Aldrich Chemie GmbH, Munich, Germany) was added to the homogenized mixture and shaken vigorously for 15 s and incubated for 3 min at RT before centrifugation at 4 °C for 15 min at 11,500 rpm. The upper aqueous phase was transferred to 2-mL sample tubes (Sarstedt, Numbrecht, Germany) and the volume adjusted to 400 µL with buffer RTL Plus (Qiagen) and loaded onto the QIAsymphony SP. All samples were eluted in 100 µL elution buffer in 96-well plates (Qiagen). Gametocyte density calibration standards were generated from tenfold serial dilution of gametocytes (10^7^–10) in 40 µL of erythrocytes.

### Molecular detection of *Plasmodium falciparum* gametocytes by RT-qPCR

*Plasmodium falciparum* gametocytes (NF54 and JH013) were quantified by RT-qPCR analysed by the LightCycler^®^ 480 Instrument II (Roche Diagnostics, Mannheim, Germany). Gametocyte-specific targets published earlier were used to measure transcript levels of *Pfs25* mRNA [22] and Pf14_0367 [23] with modifications. Custom one-step RT-qPCR assay was performed using Taqman RNA-to-C_T_™ 1-Step kit (Thermo Fisher Scientific). The reaction consisted of 1 × Taqman RT-qPCR mix, 1 × Taqman enzyme mix, 150 nM probe, and 400 nM of each primer and 2.5 µL of RNA extract. The thermal conditions consisted of reverse transcription (48 °C for 20 min), enzyme activation (96 °C for 10 min), and two-temperature cycling step (95 °C for 15 s, 62 °C for 1 min, for 45 cycles). The amplification curves were assessed for true amplification and the quantification cycle value (C_q_) was automatically calculated by the second derivative maximum method integrated in the LightCycler 480 software (version 1.5.1.62). The positivity of the amplification was defined by C_q_ value less than 40 in at least two technical replicates. The gametocyte density was derived from extrapolation of the C_q_ value obtained from the standard curve (Additional file 2: Figure S2). Primer and probe sequences are listed in Additional file 3: Table S1 and PCR conditions in Additional file 4: Table S2. All samples including the gametocyte standards were tested in triplicate in the presence of positive and negative (non-template and non-RT) controls.

### In vitro gametocytogenesis of different strains of *Plasmodium falciparum*

In order to compare the rate of gametocytogenesis of different strains of *P. falciparum*, the same culture conditions were used for gametocytes as described above. After removal of asexual parasites by the application of *N*-acetyl-d-glucosamine between days 11 and 15 of the gametocyte development, the culture was purified first by NicoPrep 1.077 cushions (AXIS-SHIELD PoC AS, Oslo, Norway) and later by LD-MACS magnetic columns (Miltenyi Biotec, Gladbach, Germany) resulting in highly purified (above 95% stage V) gametocytes (Additional file 5: Figure S3). This purification procedure was not applied to the cultures used for the longevity assay. Parasites were kept at 37 °C during the whole procedure to avoid exflagellation, as described earlier [24]. The efficiency of gametocytogenesis of three different strains of *P. falciparum* was assessed: the laboratory strains 3D7 and NF54 and 1 clinical isolate (JH013) from Lambaréné, Gabon in complete culture medium in the presence or absence of 5% serum. The parasites of all the three strains were cultured in 75-cm^2^ flasks with a starting volume of 20 mL gametocyte culture that contained 2 mL erythrocyte pellet. Gametocyte yield was determined after 15 days of culture by counting the purified stage V gametocytes using a Neubauer improved cell counting chamber. The tests were done three times in duplicate for each strain and the mean was determined.

### Statistical analysis

Descriptive statistics were calculated with GraphPad Prism version 6.0 (GraphPad Software, San Diego, CA, USA) for determining mean, percentages, standard deviation and plotting of graphs. Excel 2013 and GraphPad Prism 6 was used for the calculation of gametocytaemia of microscopic and flow cytometric data. A sample was declared positive by flow cytometry if the percentage gated cells positive for HE was above the negative control (erythrocytes cultured in complete culture medium in the incubator). PCR data were analysed by Excel and gametocytaemia was extrapolated with the help of a gametocyte standard curve with known parasitaemia. To estimate the 50 and 10% survival rate of gametocytes, Excel was used to develop a linear regression equation using the log-transformed data of the different viability assays starting on the day of highest parasitaemia. Mann–Whitney test and Wilcoxon matched-pairs signed rank test were used in GraphPad Prism 6, for non-parametric analysis of the difference in the gametocytogenesis rate among the different isolates in the presence or absence of serum.

## Results

Longevity of gametocytes was assessed by three different viability methods.

### Microscopic reading

**Fig. 1:**
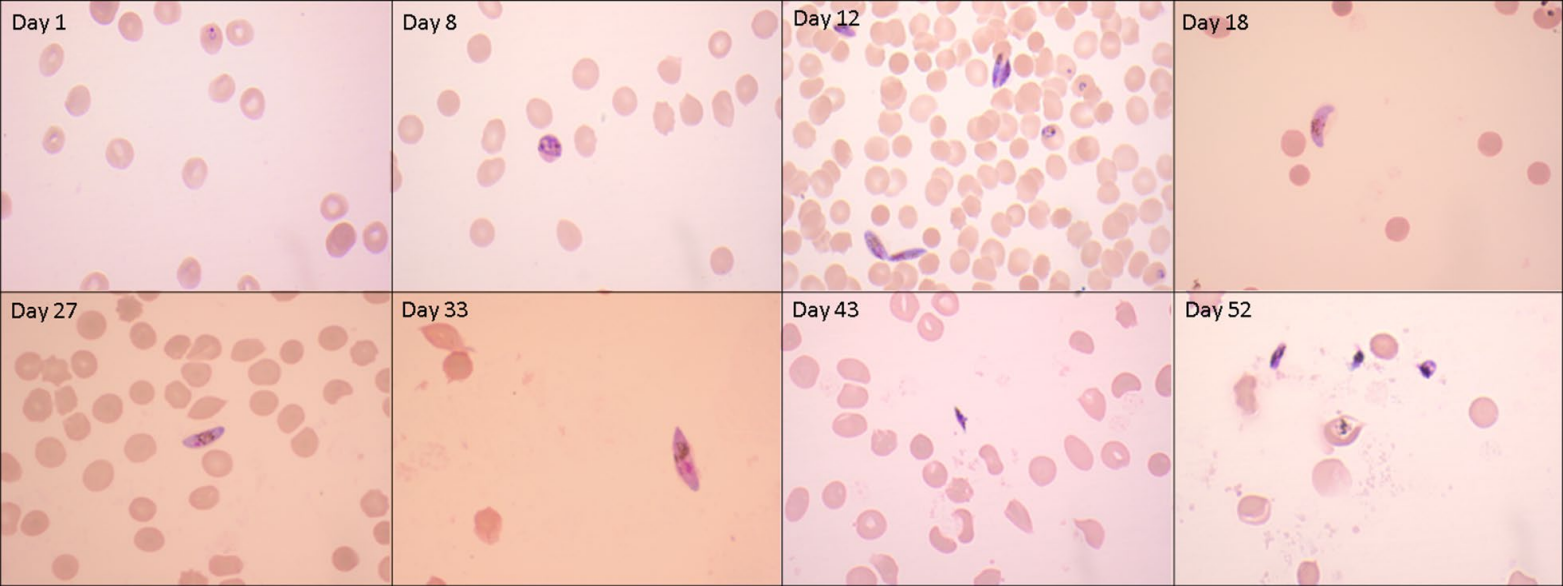
Microphotograph of Giemsa-stained thin smears of *Plasmodium falciparum* gametocyte (NF54) culture on different days of gametocyte development (magnification ×1000). *N*-Acetyl-glucosamine has been added on days 11–15 and from day 19 on every 5 days for 2 consecutive days

**Fig. 2:**
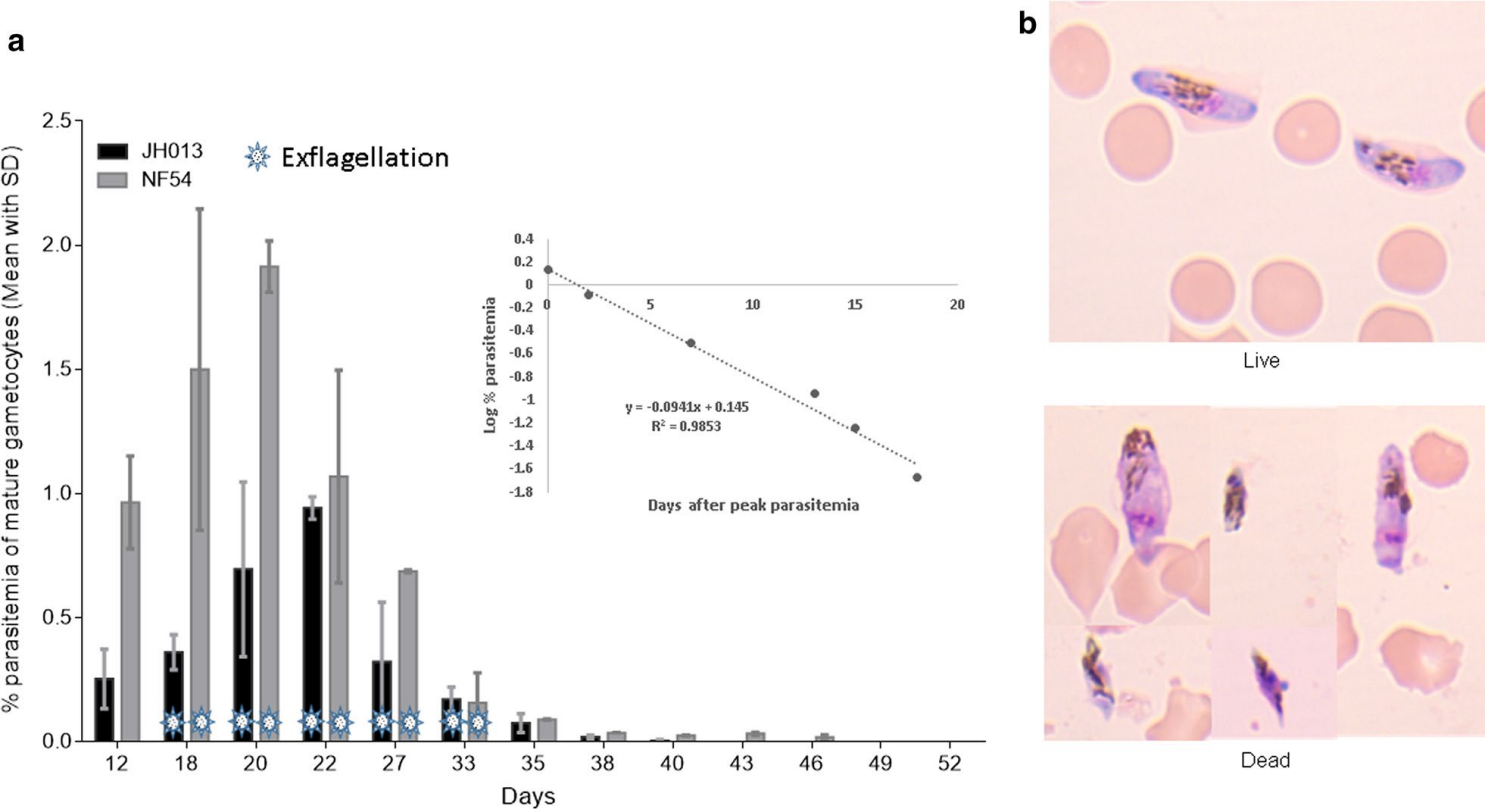
Longevity of gametocytes evaluated microscopically by morphological assessment. Viability of live gametocytes (1 clinical isolate and 1 NF54 laboratory strain) was assessed by microscopy. **a** The percentage of mature gametocytes in erythrocytes (parasitaemia) from days 12 to 52 of gametocyte culture. *Values* indicate the mean percentage (and SD) of live gametocytes counted by two qualified microscopists for the separate experiments. The days on which exflagellation was detected is indicated by a *white/blue star* at the *bottom of the bar graph*. **a** Also shows a scatter chart with a trend line and equation made using log.transformed gametocytaemia data from microscopy displayed on the y-axis of the graph. **b** Giemsa-stained thin smears of gametocyte culture with live and dead gametocytes. *Log.* logarithmic, *SD* standard deviation

The gametocytes appeared viable with intact cytoplasm for several weeks after reaching stage V (Fig. 1). Live gametocytes were detected on average for a maximum of 39 and 46 days after initiation of gametocytogenesis in JH013 and NF54, respectively. The longevity after reaching maturity was 18–26 days. The mean 50 and 10% survival rate were 2.6 and 10 days, respectively. The highest number of gametocytes can be seen on days 20–22. The percentage of live, mature, gametocyte-infected erythrocytes for the different days of the gametocyte culture evaluated by the blinded readers is shown in Fig. 2. The last viable gametocyte was seen on days 40 and 46 for JH013 and NF54 *P. falciparum* strain, respectively.

### Exflagellation assay

Exflagellation of male gametocytes was observed reproducibly until day 33, whereas from day 35 on no exflagellated males could be detected (Fig. 2). The experiment was repeated three times.

### Flow cytometry

**Fig. 3:**
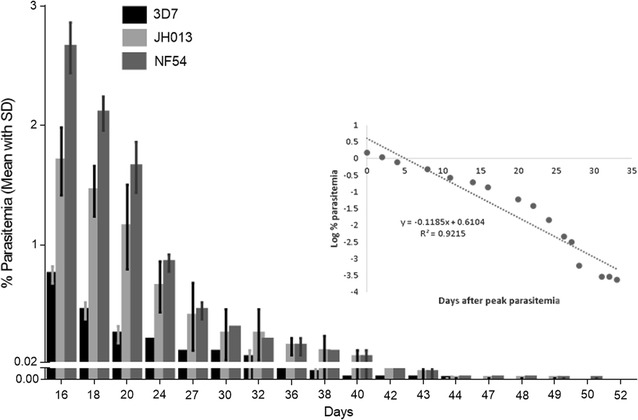
In vitro longevity of *Plasmodium falciparum* gametocytes of 3D7, NF54 and one clinical isolate (JH013) of hydroethidine (HE) stained and analysed by flow cytometry. Mean (and SD) maximum longevity of gametocytes is shown in days. Viability of gametocytes is assessed by flow cytometry measuring the percentage of positive fluorescent cells (PPFC) of 50,000 erythrocytes gated against negative control (uninfected erythrocytes stained by HE). All strains were tested at least three times. The *figure* also shows a scatter chart with a trend line and equation made using log.transformed gametocytaemia data from flow cytometry displayed on the y-axis of the graph. *Log.* logarithmic, *SD* standard deviation

Analysis of flow cytometry data measuring HE staining of *P. falciparum* gametocytes showed a longevity of 43–49 days in the tested clinical isolate and laboratory strains with an average 50% survival rate of 6.5 days, 10% survival rate of 12 days and peak gametocytaemia on day 16, following the removal of asexual parasites (Fig. 3). On day 12 there were still asexual parasites left in the culture that were also stained by HE. Therefore, these data are excluded from Fig. 3 to avoid confusion. The estimated circulation time (longevity after reaching stage V) was 16–24 days.

### Molecular detection of *Plasmodium falciparum* gametocytes by RT-qPCR

**Fig. 4:**
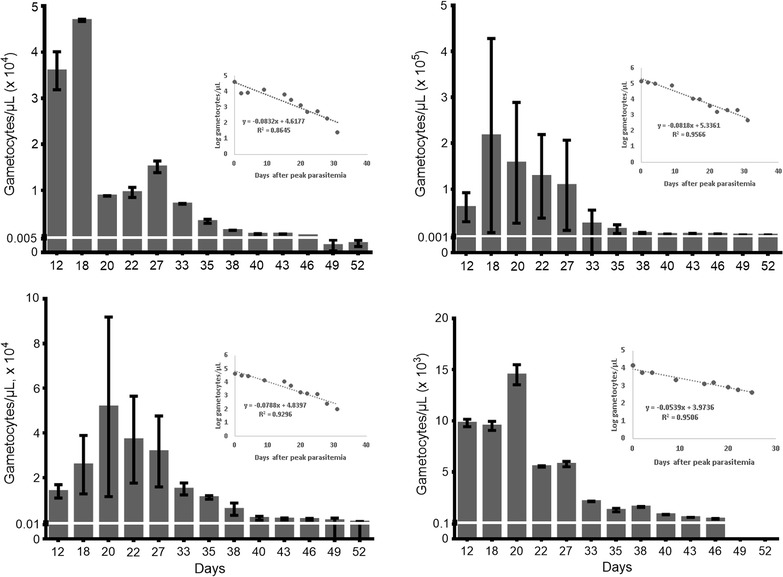
Number of gametocytes/µL of JH013 and NF54 on the different days after culture initiation measured by RT-qPCR. Quantification of test sample was done with the help of a standard curve. The previously reported gametocyte markers (PF14_0367 [23] and Pfs25 [22]) were used. Y axis shows the mean and SD. *SD* standard deviation. The figure also shows a scatter chart with a trend line and equation made using log.-transformed gametocytaemia data displayed on the y-axis of the graph. *Log.* logarithmic

RT-qPCR was used to assess a marker for mature (Pfs25 [22]) and a marker for both middle and mature stage (PF14_0367) [23]) gametocytes. The peak gametocytaemia was higher in NF54 gametocytes compared to JH013 (Fig. 4). Gametocyte longevity was longer when evaluated by PCR compared to microscopy and flow cytometry. For JH013, the PF14_0367 signal was detected until day 46 and the Pfs25 signal until day 52. Both markers were detectable until day 52 in NF54. The mature gametocyte 50 and 10% survival rate were on average 3.5 and 12 days, respectively. Peak parasitaemia was recorded on days 18 and 20 and the estimated circulation time was 31 and 26–32 days for NF54 and JH013, respectively.

### In vitro gametocytogenesis

**Fig. 5:**
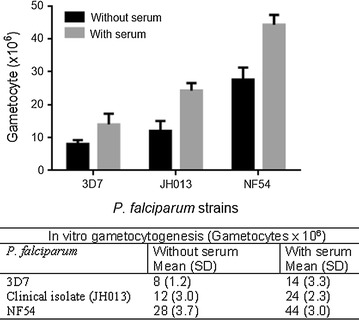
In vitro gametocytogenesis of *Plasmodium falciparum* 3D7, NF54 and JH013 cultured in complete medium in presence and absence of serum. The results shown represent the mean of at least three experiments. Mean and standard deviations are presented. The gametocyte yield was measured on day 15 after start of gametocyte culture with a starting volume of 20 mL medium with 2 mL erythrocyte pellet in 75-cm^2^ flasks. *SD* standard deviation

Rate of gametocytogenesis was assessed in three different parasite lines (3D7, NF54 and JH013) of *P. falciparum* in vitro after purifying mature gametocytes (Additional file 5: Figure S3). The highest yield of gametocytes was recorded in NF54 followed by the clinical isolate (JH013). The pattern of parasitaemia peak and decline was similar in all gametocyte cultures. Serum supplementation enhanced the gametocyte yield in all strains (Fig. 5). There was no statistically significant difference in gametocyte yield among the different strains and between the cultures with or without serum supplementation.

A summary of the longevity data evaluated by the different methods is given in Table 1, showing that analysis by RTqPCR was the most sensitive method, revealing the longest life span for gametocytes, followed by analysis by flow cytometry and microscopy. The strain NF54 had the longest life span by all methods and it was more prone for gametocyte commitment compared to the other two strains tested.

## Discussion

Density, sex ratio and life span of stage-V gametocytes of *P. falciparum* are the most important factors determining the probability of transmission to the mosquito. The maximum life span of gametocytes was explored here for the first time in vitro. Viability of gametocytes ranged from 39 to 52 days (16–32 days after reaching maturity considering peak gametocytaemia as the day of maturation) depending on the different measurement methods used, with little variability between the different parasite strains (Table 1). This time-span corresponds to the in vivo gametocyte carriage or the sum of sequestration and circulation time of the parasite in an infected person. Mature gametocytes (stage V), that freely circulate in the peripheral blood, are developed latest after 12 days [2]. Based on the ex-flagellation assay, at least 14 days of longevity was recorded for mature male gametocytes. By the protocol used here, gametocytes are generated during the first few cycles of asexual culture and therefore age of gametocytes or the day of maturity is not the same for the whole gametocyte population of one culture. However, it was observed that all gametocytes matured at the day of peak gametocytaemia, and therefore gametocyte peak was used as the day of gametocyte maturity to calculate longevity of mature gametocytes (estimated in vivo circulation time). When subtracting this maturation time, the circulation time estimated here is 16–32 days. It was observed that some gametocytes were already mature before peak gametocytaemia and the real life span or circulation time could be a bit longer than provided. In previous in vivo studies that assessed gametocytaemia by microscopy, the circulation time was estimated to be at maximum 24 [9] and 22.2 days (with mean circulation time 6.4 days) [2]. The differences in longevity to the present study likely result from the influence of host factors in in vivo studies but also from assumptions of mathematical modelling that may be incorrect, as well as the limit of detection of gametocytaemia by microscopy. It was observed in the here-presented in vitro assay that peak gametocytaemia is not yet reached on day 15, indicating the presence of some immature stages on this day. Commitment of gametocytes occurs not only on the first day of starting gametocyte culture but during the first 2–3 cycles of asexual multiplication. This might slightly affect the longevity estimate that is provided here, as some gametocytes might be some days younger than other gametocytes. To account for this the longevity of mature gametocytes (estimated circulation time) was calculated from day of peak gametocytaemia measured by the different approaches. The 50 and 10% survival rate is also calculated starting from day of peak-parasitaemia.

Another study estimated the maximum duration of gametocyte carriage (corresponding to the sum of in vivo sequestration and circulation time) to be 55 days based on molecular detection of Pfs25 and mathematical modelling [10]. Longevity of gametocytes reported in this study is similar with the current in vitro data presented here. Gametocytes were detected up to 52 days in culture by PCR when analysing the female gametocyte specific marker Pfs25. For PF14_0367 the signal was slightly weaker and in the tested clinical isolate (JH013) it was no longer detectable after day 46. The difference of the two markers is most probably due to the higher copy number and expression of Pfs25 at the gametocyte stage compared to PF14_0367 [23]. Slight differences in longevity of male and female gametocytes could also be the reason; however, no difference was seen in an earlier publication between male and female gametocyte longevity or mortality in vivo [9].

If these in vitro reared gametocytes are actually able to infect mosquitoes cannot be answered by this approach. However, in the exflagellation assay presented here, the male gametocytes were able to exflagellate for at least 2 weeks after reaching maturity. Similarly, Smalley and Sinden have reported that exflagellation of microgametes and infectivity to mosquitoes can take place for 3 weeks [9]. In line with this, a recent report showed in vivo the persistence of gametocytaemia following treatment with atovaquone–proguanil. This was later interrupted by primaquine treatment after 21 days of consistent gametocyte detection in a participant in a controlled human malaria infection trial [25].

One has to be careful to translate in vitro data to the in vivo situation as there are several contradictory factors influencing the longevity of gametocytes in vivo. On the one hand, the immune response of the host is negatively influencing longevity of the parasite, whereas on the other hand, the nutrition status might be more favourable for the parasite in the human host than in the culture flask. The in vitro culture shows longevity of gametocytes excluding the host factors.

Even though gametocytes have a long life-span in vitro, their half-life (50% survival rate) was short (2.6–6.5 days), similar to the result shown in a previous in vivo study (2.4 days) [9]. The peak gametocytaemia is followed by a steady decline in parasitaemia with few gametocytes remaining for several weeks, and around 10% surviving for 10–12 days after peak gametocytaemia. After day 40, on average less than 5% of the gametocytes remained viable. Studies have shown that if gametocytes remain viable, a small number of gametocytes (sub-microscopic level) is sufficient for transmission [7, 8]. These findings underline the importance of development of efficacious gametocidal drugs and its timely usage, especially if elimination and even eradication of malaria is the aim.

In this study, the NF54 strain of *P. falciparum* used for experimental human infection was more prone to develop into gametocytes followed by JH013 and 3D7. Similar to this report, it was shown earlier that the gametocyte yield varies between different parasite lines [26]. Many factors are considered to influence sexual commitment of the parasite including genetic variation, environmental factors [27] and long-term in vitro culture [26, 28]. The same type of medium, erythrocytes and serum (in those cultures supplemented with serum) was used to avoid modulation of gametocytogenesis by culture conditions [28].

Serum supplementation in the gametocyte culture improved the gametocyte yield in all three strains of *P. falciparum* tested. It was previously reported that serum taken from infected individuals increased the sexual conversion rate of *P. falciparum* [29]. Here it is shown that supplementation of serum from non-infected person also improves the gametocyte yield in vitro. The enhanced yield of gametocytes could also be due to an increased multiplication of the asexual parasites of the gametocyte culture when serum is used as reported earlier [29].

## Conclusions

Transmission of *P. falciparum* to mosquito depends on mature viable gametocytes in the peripheral blood of the host [30]. The in vitro life span (estimated in vivo circulation time) of mature gametocytes was 2–4 weeks in this study. These findings may help to design interventions to prevent transmission as part of elimination programs and containment of resistant *P. falciparum* isolates.

## Additional files

**Additional file 1: Figure S1.** Flow cytometry analysis of *Plasmodium falciparum* (JH013) parasites. Samples were stained with hydroethidine (HE) and analysed by flow cytometry. Panel A shows the negative control. The erythrocytes are cultured in complete culture medium and stained with HE. Panel B shows the gating strategy and percent-infected erythrocytes of a sample taken from gametocyte infected erythrocytes (from gametocyte culture).

**Additional file 2: Figure S2.** Standard curves used for gametocyte quantification by RTqPCR. Dilution series of in vitro cultivated gametocytes were analysed by PF14_0367 and Pfs25. Standard curves were generated performing linear regression using the Log_10_ transformed number of gametocytes and the mean and SEM of the quantification cycles (C_q_s) of triplicates. SEM: standard error of the mean.

**Additional file 3: Table S1.** Primer and probe sequences used in gametocyte-specific RTqPCR.

**Additional file 4: Table S2.** TaqMan One-Step Real-Time RT-PCR (RNA-to-Ct™ 1-Step Kit) PCR mix and cycling conditions.

**Additional file 5: Figure S3.** Microphotograph of Giemsa-stained thin smears showing gametocyte culture on day 15 before (A) and after (B) purification (magnification ×1000). Uninfected erythrocytes and remaining asexual parasites were removed by the two-step purifications for the in vitro measurement of gametocytogenesis of 3D7, NF54 and one clinical isolate (JH013).

## Abbreviations

Cq: quantification cycle
HE: hydroethidine
ID: identification document
PCR: polymerase chain reaction
PPFC: percentage of positive fluorescent cells
RT-qPCR: reverse transcription quantitative real-time PCR
RT: room temperature

## References

[1] WHO. World Malaria Report 2016. Geneva: World Health Organization; 2016. http://apps.who.int/iris/bitstream/10665/252038/1/9789241511711-eng.pdf?ua=1. Accessed 15 Mar 2017.

[2] Eichner M, Diebner HH, Molineaux L, Collins WE, Jeffery GM, Dietz K (2001). Genesis, sequestration and survival of *Plasmodium falciparum* gametocytes: parameter estimates from fitting a model to malariatherapy data. Trans R Soc Trop Med Hyg.

[3] Abdulsalam AH, Sabeeh N, Bain BJ (2010). Immature *Plasmodium falciparum* gametocytes in bone marrow. Am J Hematol.

[4] Aguilar R, Magallon-Tejada A, Achtman AH, Moraleda C, Joice R, Cistero P (2014). Molecular evidence for the localization of *Plasmodium falciparum* immature gametocytes in bone marrow. Blood.

[5] Alonso PL, Brown G, Arevalo-Herrera M, Binka F, Chitnis C, Collins F (2011). A research agenda to underpin malaria eradication. PLoS Med.

[6] Liu J, Modrek S, Gosling RD, Feachem RG (2013). Malaria eradication: is it possible? Is it worth it? Should we do it?. Lancet Glob Health.

[7] Churcher TS, Bousema T, Walker M, Drakeley C, Schneider P, Ouédraogo AL (2013). Predicting mosquito infection from *Plasmodium falciparum* gametocyte density and estimating the reservoir of infection. eLife.

[8] Schneider P, Bousema JT, Gouagna LC, Otieno S, van de Vegte-Bolmer M, Omar SA (2007). Submicroscopic *Plasmodium falciparum* gametocyte densities frequently result in mosquito infection. Am J Trop Med Hyg.

[9] Smalley ME, Sinden RE (1977). *Plasmodium falciparum* gametocytes: their longevity and infectivity. Parasitology.

[10] Bousema T, Okell L, Shekalaghe S, Griffin JT, Omar S, Sawa P (2010). Revisiting the circulation time of *Plasmodium falciparum* gametocytes: molecular detection methods to estimate the duration of gametocyte carriage and the effect of gametocytocidal drugs. Malar J.

[11] Lelièvre J, Almela MJ, Lozano S, Miguel C, Franco V, Leroy D (2012). Activity of clinically relevant antimalarial drugs on *Plasmodium falciparum* mature gametocytes in an ATP bioluminescence “transmission blocking” assay. PLoS ONE.

[12] Butterworth AS, Skinner-Adams TS, Gardiner DL, Trenholme KR (2013). *Plasmodium falciparum* gametocytes: with a view to a kill. Parasitology.

[13] Diebner HH, Eichner M, Molineaux L, Collins WE, Jeffery GM, Dietz K (2000). Modelling the transition of asexual blood stages of *Plasmodium falciparum* to gametocytes. J Theor Biol.

[14] Sutherland CJ, Ord R, Dunyo S, Jawara M, Drakeley CJ, Alexander N (2005). Reduction of malaria transmission to Anopheles mosquitoes with a six-dose regimen of co-artemether. PLoS Med.

[15] Drakeley CJ, Jawara M, Targett GAT, Walraven G, Obisike U, Coleman R (2004). Addition of artesunate to chloroquine for treatment of *Plasmodium falciparum* malaria in Gambian children causes a significant but short-lived reduction in infectiousness for mosquitoes. Trop Med Int Health.

[16] Bousema T, Sutherland CJ, Churcher TS, Mulder B, Gouagna LC, Riley EM (2011). Human immune responses that reduce the transmission of *Plasmodium falciparum* in African populations. Int J Parasitol.

[17] Ouédraogo AL, Bousema T, Schneider P, de Vlas SJ, Ilboudo-Sanogo E, Cuzin-Ouattara N (2009). Substantial contribution of submicroscopical *Plasmodium falciparum* gametocyte carriage to the infectious reservoir in an area of seasonal transmission. PLoS ONE.

[18] Held J, Westerman R, Kremsner PG, Mordmüller B (2010). In vitro activity of mirincamycin (U24729A) against *Plasmodium falciparum* isolates from Gabon. Antimicrob Agents Chemother.

[19] Mordmüller B, Supan C, Sim KL, Gómez-Pérez GP, Salazar CLO, Held J (2015). Direct venous inoculation of *Plasmodium falciparum* sporozoites for controlled human malaria infection: a dose-finding trial in two centres. Malar J.

[20] Ghosh AK, Dinglasan RR, Ikadai H, Jacobs-Lorena M (2010). An improved method for the in vitro differentiation of *Plasmodium falciparum* gametocytes into ookinetes. Malar J.

[21] Chevalley S, Coste A, Lopez A, Pipy B, Valentin A (2010). Flow cytometry for the evaluation of anti-plasmodial activity of drugs on *Plasmodium falciparum* gametocytes. Malar J.

[22] Wampfler R, Mwingira F, Javati S, Robinson L, Betuela I, Siba P (2013). Strategies for detection of Plasmodium species gametocytes. PLoS ONE.

[23] Joice R, Narasimhan V, Montgomery J, Sidhu AB, Oh K, Meyer E (2013). Inferring developmental stage composition from gene expression in human malaria. PLoS Comput Biol.

[24] Gebru T, Mordmuller B, Held J (2014). Effect of fluorescent dyes on in vitro-differentiated, late-stage *Plasmodium falciparum* gametocytes. Antimicrob Agents Chemother.

[25] Hanron AE, Billman ZP, Seilie AM, Olsen TM, Fishbaugher M, Chang M (2017). Multiplex, DNase-free one-step reverse transcription PCR for Plasmodium 18S rRNA and spliced gametocyte-specific mRNAs. Malar J..

[26] Graves PM, Carter R, McNeill KM (1984). Gametocyte production in cloned lines of *Plasmodium falciparum*. Am J Trop Med Hyg.

[27] Dyer M, Day KP (2000). Commitment to gametocytogenesis in *Plasmodium falciparum*. Parasitol Today.

[28] Carter R, Miller LH (1979). Evidence for environmental modulation of gametocytogenesis in *Plasmodium falciparum* in continuous culture. Bull World Health Organ.

[29] Smalley ME, Brown J (1981). *Plasmodium falciparum* gametocytogenesis stimulated by lymphocytes and serum from infected Gambian children. Trans R Soc Trop Med Hyg.

[30] Da DF, Churcher TS, Yerbanga RS, Yaméogo B, Sangaré I, Ouedraogo JB (2015). Experimental study of the relationship between *Plasmodium gametocyte* density and infection success in mosquitoes; implications for the evaluation of malaria transmission-reducing interventions. Exp Parasitol.

