# Supplementary figures and images for: Life-span of in vitro differentiated Plasmodium falciparum gametocytes
Source: Malar J. 2017 Aug 11;16:330. doi: 10.1186/s12936-017-1986-6 (PMC5553604; doi:10.1186/s12936-017-1986-6)

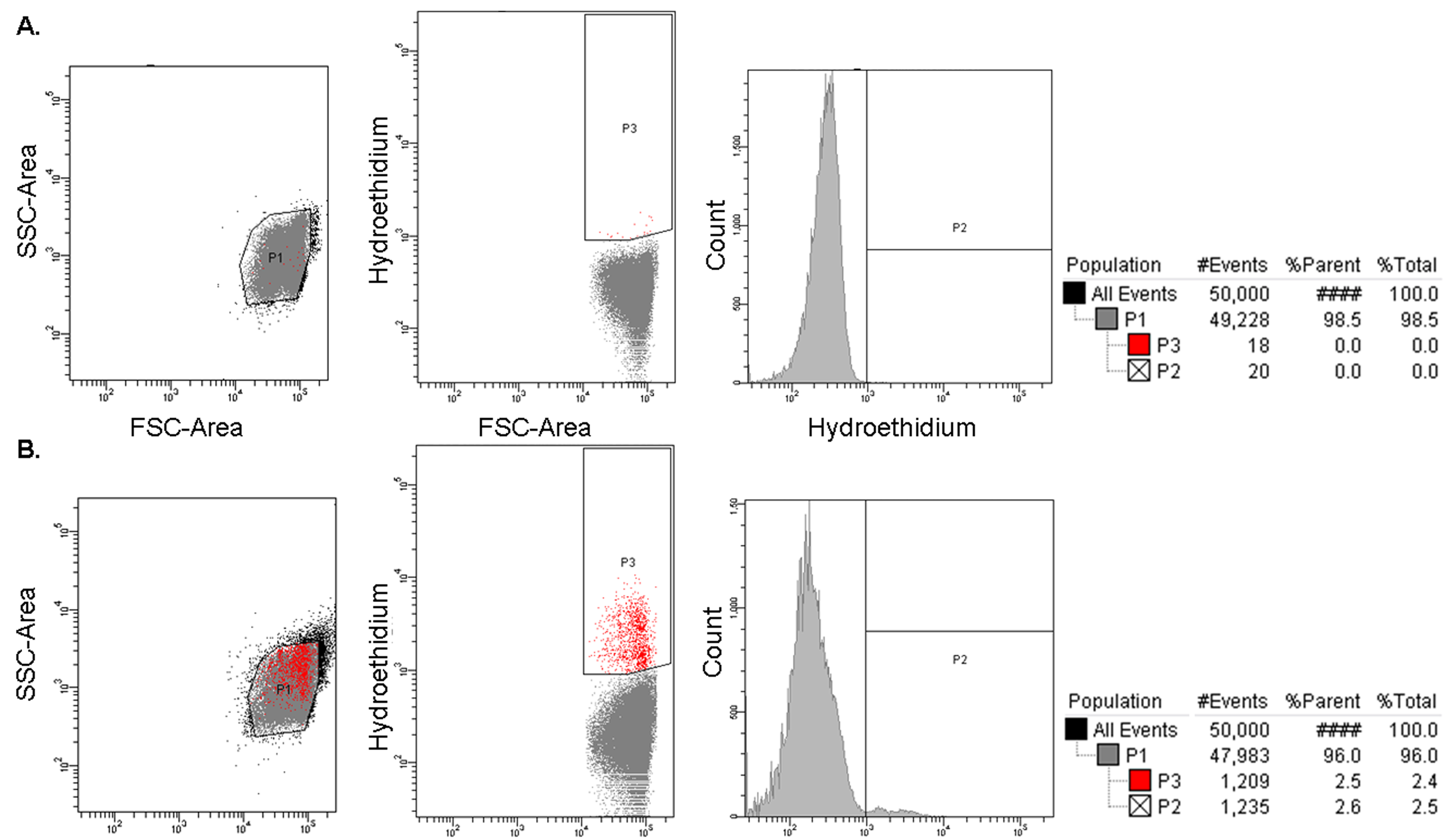

Supplement: Supplementary file 1 — Additional file 1: Figure S1. Flow cytometry analysis of Plasmodium falciparum (JH013) parasites. Samples were stained with hydroethidine (HE) and analysed by flow cytometry. Panel A shows the negative control. The erythrocytes are cultured in complete culture medium and stained with HE. Panel B shows the gating strategy and percent-infected erythrocytes of a sample taken from gametocyte infected erythrocytes (from gametocyte culture). [file 12936_2017_1986_MOESM1_ESM.tif]

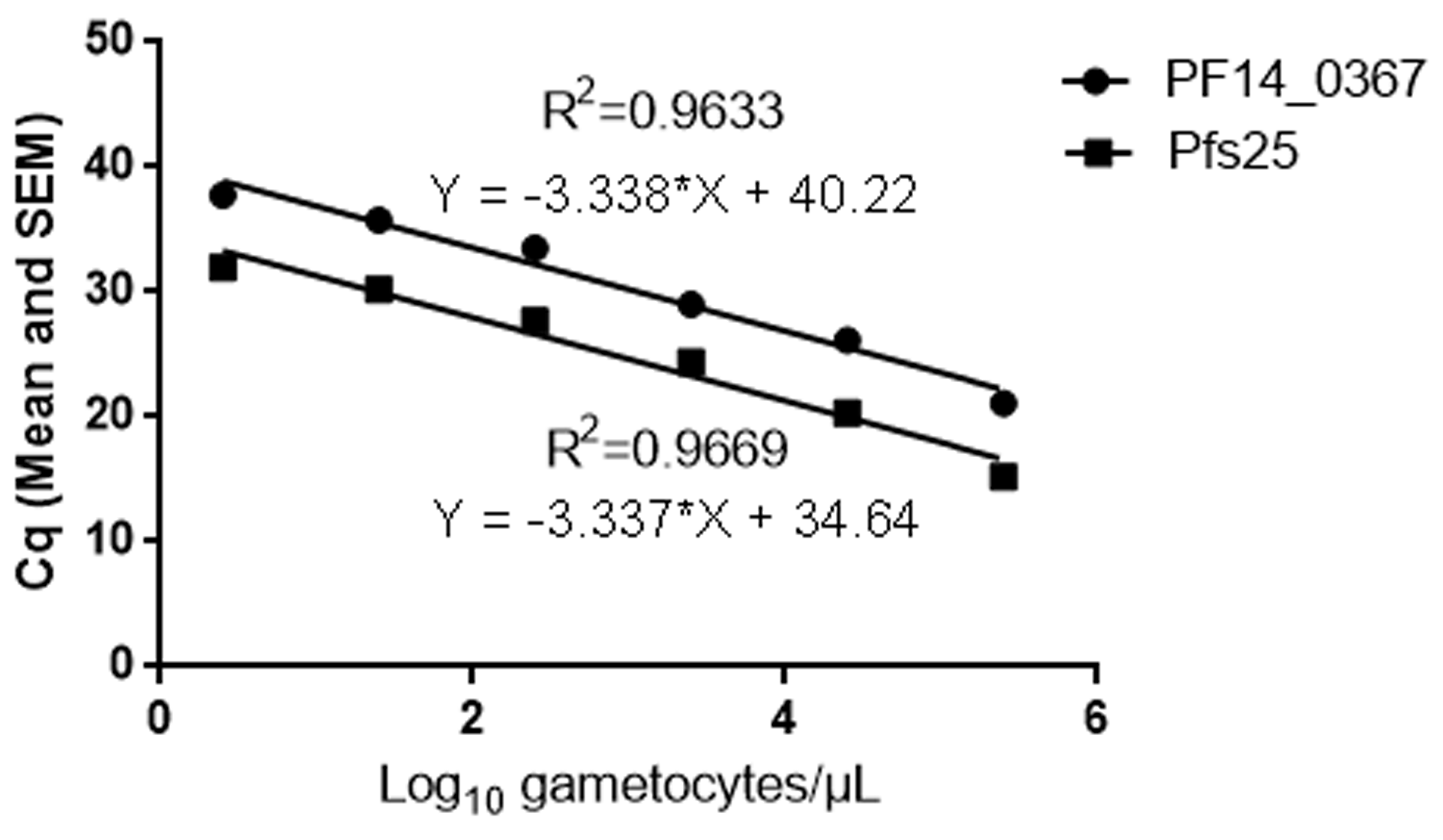

Supplement: Supplementary file 2 — Additional file 2: Figure S2. Standard curves used for gametocyte quantification by RTqPCR. Dilution series of in vitro cultivated gametocytes were analysed by PF14_0367 and Pfs25. Standard curves were generated performing linear regression using the Log10 transformed number of gametocytes and the mean and SEM of the quantification cycles (Cqs) of triplicates. SEM: standard error of the mean. [file 12936_2017_1986_MOESM2_ESM.tif]

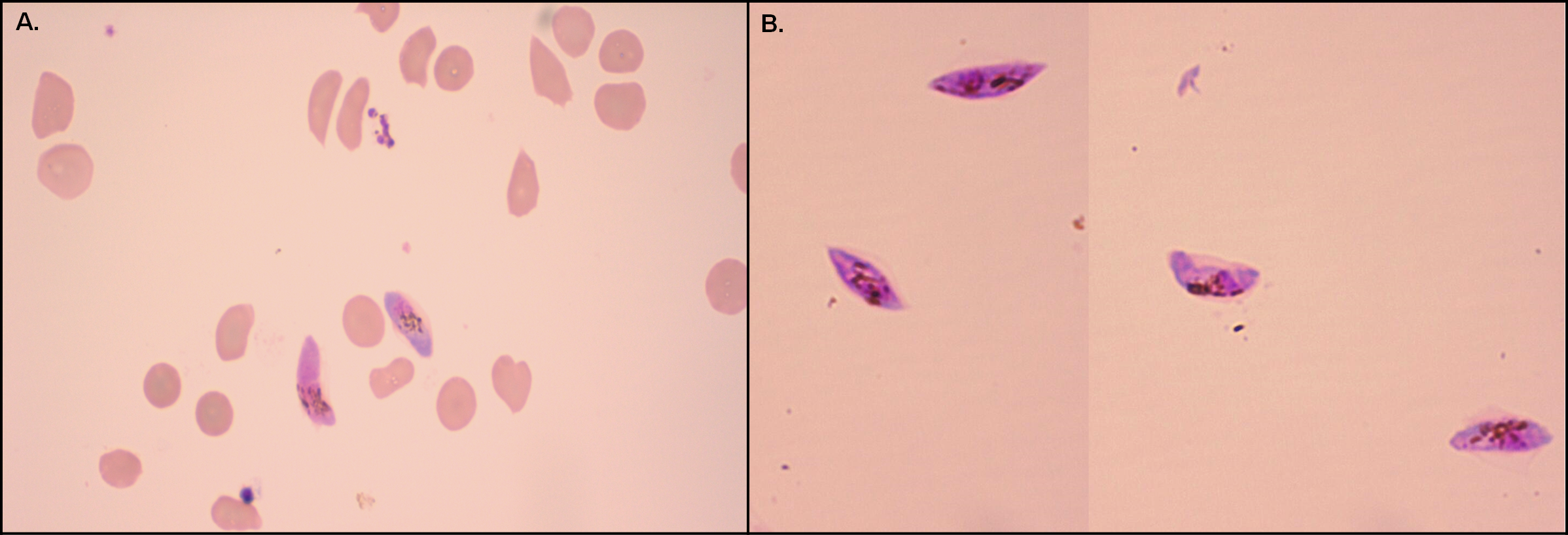

Supplement: Supplementary file 5 — Additional file 5: Figure S3. Microphotograph of Giemsa-stained thin smears showing gametocyte culture on day 15 before (A) and after (B) purification (magnification ×1000). Uninfected erythrocytes and remaining asexual parasites were removed by the two-step purifications for the in vitro measurement of gametocytogenesis of 3D7, NF54 and one clinical isolate (JH013). [file 12936_2017_1986_MOESM5_ESM.tif]
