# Supplementary material for: Life-span of in vitro differentiated Plasmodium falciparum gametocytes
Source: Malar J. 2017 Aug 11;16:330. doi: 10.1186/s12936-017-1986-6 (PMC5553604; doi:10.1186/s12936-017-1986-6)
Supplement: Supplementary file 3 — Additional file 3: Table S1. Primer and probe sequences used in gametocyte-specific RTqPCR. [file 12936_2017_1986_MOESM3_ESM.docx]

**Table S1. Primer and probe sequences used in gametocyte-specific RTqPCR.**

| **Species** | **Target gene** | **Primer name** | **Primer sequence (5’->3’)** |
| --- | --- | --- | --- |
| ***P. falciparum*** | **Pfs25** ^1^ | Pfs25_Fwd | GAC TGT AAA TAA ACC ATG TGG AGA |
|  |  | Pfs25_Rev | CAT TTA CCG TTA CCA CAA GTT A |
|  |  | Pfs25_Probe | LC640 - AGA TGG AAA TCC CGT TTC ATA CGC TTG T |
|  | ***PF14_0367*** ^1^ *(*mid-late gametocytes) | MG_Fwd | GTTACATTTCGACCCAGCATAAATT |
|  |  | MG_Rev | GTTACATTTCGACCCAGCATAAATT |
|  |  | MG_Probe | VIC - CAG TGC ATA TTG TTG CCT GT - MGBNFQ |

^1^ The primer and probe sequences of mid-late stage gametocyte marker (PF14_0367) were previously published (Joice *et al* 2013), whereas we have designed a new sequences for the Pfs25 marker.
