# Supplementary material for: Life-span of in vitro differentiated Plasmodium falciparum gametocytes
Source: Malar J. 2017 Aug 11;16:330. doi: 10.1186/s12936-017-1986-6 (PMC5553604; doi:10.1186/s12936-017-1986-6)
Supplement: Supplementary file 4 — Additional file 4: Table S2. TaqMan One-Step Real-Time RT-PCR (RNA-to-Ct™ 1-Step Kit) PCR mix and cycling conditions. [file 12936_2017_1986_MOESM4_ESM.doc]

**Table S2**. **TaqMan One-Step Real-Time RT-PCR (**[**RNA-to-Ct™ 1-Step Kit**](https://www.thermofisher.com/de/de/home/life-science/pcr/real-time-pcr/real-time-pcr-reagents/one-step-real-time-rt-master-mix/taqman-rna-to-ct-1-step.html?ICID=cvc-qpcr-onestep-c1t1)**) PCR mix and cycling conditions.**

1. **RT-qPCR Reaction Mix** ^1^

| **PCR mix** | **Volume (µL)** |
| --- | --- |
| TaqMan® RT-PCR Mix (2✕) | 5 |
| Forward primer | 0.4 |
| Reverse primer | 0.4 |
| TaqMan probe | 0.15 |
| RNA template | 2.5 |
| RNase-free H2O | 1.3 |
| TaqMan® RT Enzyme Mix (40✕) | 0.25 |
| Total | 10 |

1. **RT-qPCR Thermal cycling conditions** ^2^

| **Stage** | **Step** | **Temperature** | **Time** |
| --- | --- | --- | --- |
| Holding | Reverse transcription | 48°C | 20 minutes |
| Holding | Polymerase activation | 96°C | 10 minutes |
| Cycling (45x) | Denaturation | 95°C | 15 seconds |
|  | Annealing/Extension | 62°C | 1 minute |
| Holding | Cooling | 48°C | 10 seconds |

^1^ The reaction was prepared in a PCR cabinet and the mix was dispensed to the 384 wells using a Qiagen QIAgility High-Precision Automated PCR System.

^2^ The PCR was run using the [LightCycler® 480 Real-Time PCR System](https://www.google.de/url?sa=t&rct=j&q=&esrc=s&source=web&cd=2&cad=rja&uact=8&ved=0ahUKEwiLloDs4cnRAhVoJ5oKHWddD9oQFggsMAE&url=http%3A%2F%2Fwww.gene-quantification.de%2Flc480-brochure.pdf&usg=AFQjCNEcni7j_Jz2Vqjc7uVj641nB_ziVA&sig2=8pS8FI5LTXuZVwfJx9gTYw) (Roche Diagnostics, Mannheim, Germany) and all samples with Ct value ≤40 were considered positive.
